# Suicidal ideation in adolescents and their caregivers: a cross sectional survey in Japan

**DOI:** 10.1186/s12888-016-0934-2

**Published:** 2016-07-11

**Authors:** Kentaro Kawabe, Fumie Horiuchi, Marina Ochi, Yasunori Oka, Shu-ichi Ueno

**Affiliations:** Department of Neuropsychiatry, Ehime University Graduate School of Medicine, Toon City, Ehime Japan; Center for Child Health, Behavior and Development, Ehime University Hospital, Toon City, Ehime Japan; Center for Sleep Medicine, Ehime University Hospital, Toon City, Ehime Japan

**Keywords:** Adolescent, Caregivers, General Health Questionnaire (GHQ), Profile of Mood States (POMS), Suicidal ideation, Suicide prevention

## Abstract

**Backgrounds:**

Suicide is a leading cause of death in adolescence. Effective strategies are required to prevent suicide. We aimed to assess the prevalence of suicidal ideation in early teens and the relationship between family mental health and suicidal ideation of their child.

**Methods:**

A population-based survey in a rural town included 185 junior high school students and their caregivers. Suicidal ideation and mental states were assessed with General Health Questionnaire (GHQ) and Profile of Mood States (POMS) form.

**Results:**

Nineteen (10.3 %) students experienced suicidal ideation in the preceding weeks and had more mental health problems than students without suicidal ideation. Caregivers of students with suicidal ideation demonstrated significantly higher suicidal depression scores in GHQ. Multivariate logistic regression analysis revealed that suicidal depression of caregivers was the most important factor for suicidal ideation of students.

**Conclusions:**

Suicidal ideation of children is associated with suicidal depression of their caregivers. For the prevention of suicide in adolescents, not only their own mental status but also that of caregivers should be taken into consideration.

## Background

Suicide is a major health problem among adolescents, and adolescent suicide is currently the third leading cause of death, accounting for 10 % of all deaths in those aged 15-19 [1]. Suicide rates increase ten-fold from preadolescence to early adulthood [2]. According to the Japanese 2009 Vital Statistics Report, the highest number of deaths in the 15–39 year age group was attributed to suicide [3], and the suicide rate of Japanese adolescents is consistent with international suicide rates. Moreover, the suicide rate in 15–19 year olds, which was 4 per 100,000 populations in 1990, had approximately doubled by 2010. Adolescent suicide is a major public health concern and an important psychiatric condition with significant implications for mental health service planning. Thus, prevention of suicide is one of the most important mental health issues in adolescents [4]. Although the causes are complex, the aim of suicide prevention is to reduce risk factors and promote resilience or coping. Prevention should occur at all levels of the society, i.e., from the individual, family, and community levels.

Although suicide prevention is major political issue in many countries, significance of the problem differs among the counties. For example, number of suicidal death exceeded thirty thousand for many years in Japan, the Japanese Cabinet Office released in 2006 the ‘General Principles of Suicide Prevention Policy’, which aimed at reducing the overall suicide rate by 20 % over the next decade. Additionally, the Cabinet Office launched a national fund to help local authorities implement suicide-prevention programs [5, 6].

The national fund is allocated to each prefecture and municipal, however, suicide rate is different from each prefecture and municipal. The rate of suicide in Kumakogen town, in which we conducted this study, was the highest among the region, therefore, the town was selected as one of the areas funded for suicide prevention. The suicide prevention program targeted especially at the elderly during the first phase, and the target moved to adolescents afterwards. We have been involved in the project for adolescent suicide prevention under the cooperation with medical professionals and local authorities in the town [7]. In order to achieve suicide prevention among the community, handy method of identifying effective target of intervention need to be established.

Psychiatric problems are important risk factors for suicide and considered as the effective target of intervention. A systematic review of psychological autopsy studies of completed suicides indicates that 87.3 % of cases have a history of psychiatric disorders [8]. Mood disorders, substance abuse, and prior suicidal attempts are strongly correlated with suicides in youth. Family problems, social alienation, and other precipitating problems also contribute to suicide risk [9]. Furthermore, a family history of completed suicides or psychiatric illnesses is a risk factor for suicide [10]. An association between mental states in adolescents and family factors has previously been reported [11]. Susukida et al. reported that perceived support from caregivers during childhood and lifetime suicidal ideation was associated regardless of family structure [12]. On the other hand, suicidal behaviour and depression in parents are specifically correlated with suicidal behaviour in their children [13]. The family mental health would be one of the causal factors.

The present study, therefore, investigated the effective way of intervention in the relationship of suicidal ideation and mental health between adolescents (aged 12–15 years) and their caregivers. We hypothesized that the familial risk factors would be identified specifically through comparable batteries of questions. The aim of this study was to identify the prevalence of suicidal ideation in early teens and the way of assessment in the relationship between family mental health and suicidal ideation of their child.

## Methods

### Participants

This is a cross-sectional survey conducted in Kumakogen Town (population: 9,600), a rural region of Ehime Prefecture (population: 1,390,000) located in southwest Japan. Three public junior high school students participated in the study. Three grades of junior high school students (12–15 years old) were included except mentally retarded (Intelligence Quotient: IQ below 70) or physically handicapped students. The mental states of both junior high school students and their parents or primary caregivers were investigated in this study. The study was approved by the Institutional Review Board of Ehime University Graduate School of Medicine and the School Board of Kumakogen Town. The study protocol was explained to the junior high school teachers of the area. Students were informed about the questionnaires being used in the study. Primary caregivers were informed of this study by letter and asked to contact the school for any clarification. In September 2012, students completed the questionnaires at school, whereas their primary caregivers completed the questionnaires at home. Written informed consent was obtained from each student and caregiver of them.

### Evaluation of mental state and suicidal ideation

The mental states of students and their caregivers were similarly assessed with the 30-item Japanese version of the General Health Questionnaire (GHQ), which was validated in Japan for youth aged over 12 years [14–16]. GHQ assesses general psychopathology and measures mental state using six scales: general illness, somatic symptoms, sleep disturbance, social, anxiety and dysphoria and suicidal depression that were summed to give a total score. Respondents are asked to rate each item according to the degree it was applicable during the preceding 2 or 3 weeks. Two scoring systems were used, the Likert scale and two-point scale. In the Likert scale, the four possible answers ‘better than usual’, ‘same as usual’, ‘worse than usual’ and ‘much worse than usual’ were assigned scores of 0, 1, 2 and 3 points, respectively. The minimum possible total score for each scale was 0, and the maximum possible was 15. In this study, Cronbach’s alpha coefficient for the Likert scale scoring was 0.90 for the students’ responses and 0.92 for those of the caregivers.

Suicidal ideation was assessed by item 30 of GHQ: ‘Thoughts of taking your life?’ This question inquiries about active thoughts of killing oneself that is in line with the Columbia Classification Algorithm of Suicide Assessment [17]. Students with a score >2 for item 30 were classified as having suicidal ideation.

### Assessment of mood

Mood was assessed using the Japanese edition of the Profile of Mood States Brief Form (POMS) [18]. The POMS is a 30-item self-report questionnaire to assess psychological distress, with higher levels of reliability and validity for those aged over 15 years. Moreover, POMS has been used for young people above 12 years in previous studies [19, 20]. The POMS measures mood using six subscales: tension–anxiety, depression–dejection, anger–hostility, vigour–activity, fatigue–inertia and confusion–bewilderment, and the POMS total mood disturbance (TMD) score is calculated by summing the scores across all six factors by negatively weighing vigour–activity. Respondents are asked to rate the degree to which an adjective applied to them during the preceding week on a 5-point scale (0 = ‘not at all’ and 4 = ‘extremely’). Scores for each subscale range from 0 to 20; higher scores indicate more severe symptoms except for the vigour–activity scale, where lower scores indicate more severe symptoms [21]. Cronbach’s alpha coefficients for POMS scores for students and their caregivers were 0.92 and 0.92, respectively.

### Statistical analysis

Descriptive statistics (mean, standard deviation, and percentage) were calculated to reflect the backgrounds of the participants. To compare the mental health of students and their caregivers, the mean scores of GHQ and POMS were analysed using Wilcoxon signed-rank test. The mean scores of GHQ and POMS among students and caregivers were compared using Mann–Whitney U-test between students with and without suicide ideation.

To elucidate the main factor that affects the suicidal ideation of students, we first performed a series of univariate logistic regression analyses individually. The variables used for adjustment were sex, grade, subscales of POMS and GHQ of students and caregivers. Variables found to be significant in univariate analyses were then subjected to multivariable logistic regression analysis with a backwards stepwise regression procedure. To be able to conduct a multivariable logistic regression analysis, the outcome of GHQ and POMS were divided into independent variables: 0 = below a cut-off point and 1 = over a cut-off point. In GHQ, a cut-off point of 3 or higher was used to classify in the subscale of general illness, somatic symptoms, sleep disturbance, and social dysfunction; 4 or higher was used in anxiety and dysphoria; and 2 or higher was used in suicidal depression [22]. The subscale of suicidal depression in GHQ include suicidal ideation item, Therefore, the GHQ suicidal depression subscale of students was not included in the regression analysis as an independent variable. In POMS, a cut-off point for T-scores, 60 and above was scored 1 and less than 60 was scored 0, because T-scores were calculated by subscale and cut-off points for each total and subscale were 60 and above [23]. All statistical analyses were performed using the SPSS Statistics (IBM Corp., Armonk, NY, USA) for Windows, Version 22.0. The significance level was set at *p* < 0.05, and all tests were 2-tailed.

## Results

### Demographic data

All 220 students aged 12–15 years (77, first grade; 66, second grade and 77, third grade) filled out their questionnaires. Seventeen (7.7 %) of the 220 caregivers did not participate in this study, and 18 (8.2 %) responses with one or more missing data on questionnaire were excluded. Finally, a total of 185 students (90 males and 95 females) and their caregivers (154 mothers, 25 fathers and six others) were included in this study (final response rate, 84.1 %). The median age, GHQ and POMS scores of students and their caregivers are listed in Table 1. The scores for general illness and sleep disturbance of GHQ of the caregivers were significantly higher than those of students. There were no statistical differences of the GHQ and POMS scores between 185 students included in the further study and 35 students who were not included in the further study.

**Table 1:** Baseline characteristics of the participants

	Students (*n* = 185)		Caregivers (*n* = 185)			
	*n* (%)		*n* (%)			
Sex						
Male	90 (48.6)					
Female	95 (51.4)					
Grade						
First	61 (33.0)					
Second	58 (31.4)					
Third	66 (35.7)					
Caregiver						
Mother			154 (83.2)			
Father			25 (13.5)			
Other			6 (3.2)			
	Median (quartile)	95 % CI	Median (quartile)	95 % CI	*Z*	*P*
Age, years	14 (13, 14)	13.45-13.73	43 (40, 46)	42.09-43.61		
GHQ score,						
General illness	4 (3, 5)	3.70-4.33	5 (3, 7)	4.91-5.74	4.82	**<0.01**
Somatic symptoms	4 (1, 6)	3.56-4.44	4 (1.5, 6.5)	3.74-4.64	0.63	0.53
Sleep disturbance	2 (1, 5)	2.97-3.86	4 (2, 8)	4.43-5.47	4.53	**<0.01**
Social dysfunction	5 (4, 6)	4.47-5.01	5 (4, 5)	4.72-5.24	1.15	0.25
Anxiety and dysphoria	3 (1, 6)	3.45-4.45	4 (2, 6)	3.79-4.76	1.09	0.28
Suicidal depression	0 (0, 4)	1.81-2.80	0 (0, 4)	1.52-2.34	1.18	0.24
POMS score						
Total mood disturbance	17 (7, 29)	18.04-23.19	16 (8, 30)	17.76-22.64	0.19	0.85
Tension–anxiety	2 (1, 6)	3.51-4.70	4 (1, 6.5)	3.79-4.87	0.93	0.35
Depression–dejection	2 (0, 6)	3.06-4.17	2 (0, 4)	2.42-3.40	1.94	0.05
Anger–hostility	3 (1, 6)	3.64-4.84	4 (2, 6)	4.23-5.36	1.96	0.05
Vigor	5 (2, 7)	4.49-5.59	4 (2, 7)	3.96-4.87	1.0	0.32
Fatigue–inertia	6 (3, 10)	6.44-7.94	6 (3, 9.5)	5.97-7.38	0.85	0.40
Confusion–bewilderment	6 (4, 9)	6.01-7.00	5 (4, 8)	5.48-6.33	1.7	0.09

Wilcoxon signed-rank test was used for statistics. Significant data are shown in bold

*CI* confidence interval, *GHQ* the 30-item version of the general health questionnaire, *POMS* the profile of mood states brief form

### Prevalence of suicidal ideation

Out of 185 students and caregivers, 19 students (male: female = 9:10) and five caregivers (mother: father: other = 3:1:1) indicated suicidal ideation by a score >2 for GHQ item 30. In students, the overall prevalence of suicidal ideation was 10.3 % (95 % confidence interval 5.9 %–14.6 %), with 11.1 % (4.6 %–17.6 %) in males and 9.5 % (3.6 %–15.4 %) in females. In caregivers, the overall prevalence of suicidal ideation was 2.7 % (0.4 %–5.0 %).

### Comparison of mental states between students with and without suicidal ideation

The POMS TMD was significantly higher in students with suicidal ideation than in students without suicidal ideation (Table 2). The subscale scores of GHQ for sleep disturbance, anxiety and dysphoria and suicidal depression and those of POMS for tension–anxiety, depression–dejection, anger–hostility, fatigue–inertia and confusion–bewilderment were significantly higher in students with suicidal ideation.

**Table 2:** Comparison of GHQ and POMS scores between students with and without suicidal ideation

	Students without suicidal ideation (n = 166)		Students with suicidal ideation (n = 19)		*U*	*P*
	*n* (%)		*n* (%)			
Sex						
Male	80 (48.2)		10 (52.6)			
Grade						
First	57 (34.3)		4 (21.0)			
Second	49 (29.5)		9 (47.4)			
Third	60 (36.1)		6 (31.6)			
	Median (quartile)	95 % CI	Median (quartile)	95 % CI		
Age, years	14 (13, 14)	13.43-13.72	14 (13, 15)	13.21-14.27	1424.5	0.47
GHQ score						
General illness	4 (3, 5)	3.73-4.41	3 (2, 4)	2.77-4.29	1334.5	0.27
Somatic symptoms	4 (1, 6)	3.40-4.31	5 (2, 8)	3.58-6.95	1199.0	0.09
Sleep disturbance	2 (1, 5)	2.82-3.74	4 (2, 6)	3.12-6.14	1127.5	**0.04**
Social dysfunction	5 (4, 6)	4.49-5.04	4 (3, 6)	3.49-5.56	1444.0	0.54
Anxiety and dysphoria	3 (1, 6)	3.12-4.11	7 (3, 10)	4.95-8.74	832.0	**<0.01**
Suicidal depression	0 (0, 3)	1.18-1.92	10 (6, 11)	7.13-10.66	161.0	**<0.01**
POMS score						
Total mood disturbance	15 (7, 28)	16.22-21.14	34 (20, 55)	26.61-48.44	839.5	**<0.01**
Tension–anxiety	2 (1, 6)	3.14-4.31	7 (2, 12)	4.97-9.88	899.5	**<0.01**
Depression–dejection	2 (0, 5)	2.74-3.85	7 (3, 9)	4.23-8.61	905.0	**<0.01**
Anger–hostility	3 (1, 5.3)	3.25-4.38	6 (4, 12)	5.15-10.75	870.0	**<0.01**
Vigor	5 (2, 7.3)	4.52-5.70	4 (2, 7)	2.89-5.96	1440.0	0.53
Fatigue–inertia	6 (3, 10)	6.06-7.54	9 (4. 16)	7.34-13.82	1065.5	**0.02**
Confusion–bewilderment	5 (4, 8)	5.69-6.63	10 (5, 12)	7.35-11.81	867.0	**<0.01**

Mann–Whitney U test was used for statistics. Significant data are shown in bold

*CI* confidence interval, *GHQ* the 30-item version of the general health questionnaire, *POMS* the profile of mood states brief form

### Comparison of mental states of caregivers between students with and without suicidal ideation

Mental states were compared between 19 caregivers whose children had suicidal ideation (5 fathers, 12 mothers, and 2 other caregivers) and the remaining 166 caregivers (Table 3). The GHQ subscale score for suicidal depression was significantly higher for caregivers of students with suicidal ideation than for those without suicidal ideation (Table 3).

**Table 3:** Comparison of GHQ and POMS scores of caregivers between students with and without suicidal ideation

	Caregivers of students without suicidal ideation (*n* = 166)		Caregivers of students with suicidal ideation (*n* = 19)		*U*	*P*
	Median (quartile)	95 % CI	Median (quartile)	95 % CI		
Age, years	43 (39.3, 46)	41.90-43.49	44 (40.5, 48)	41.50-47.09	1139.5	0.27
GHQ score						
General illness	5 (3, 7)	4.88-5.75	5 (3, 8)	3.85-6.99	1557.5	0.93
Somatic symptoms	4 (1, 7)	3.74-4.71	3 (2, 5)	2.59-5.20	1507.5	0.75
Sleep disturbance	4 (2, 8)	4.38-5.49	5 (3, 8)	3.42-6.69	1504.0	0.74
Social dysfunction	5 (4, 5)	4.60-5.08	5 (5, 7)	4.66-7.66	1172.0	0.05
Anxiety and dysphoria	4 (2, 6)	3.66-4.62	4 (0, 10)	3.18-7.76	1390.5	0.40
Suicidal depression	0 (0, 3)	1.23-1.94	5 (1, 10)	2.90-7.09	813.0	**<0.01**
POMS score						
Total mood disturbance	15 (8, 30)	16.89-21.87	25 (8, 44)	17.52-37.22	1219.5	0.11
Tension–anxiety	4 (1, 6)	3.62-4.75	6 (2, 9)	3.55-7.61	1250.0	0.14
Depression–dejection	2 (0, 4)	2.25-3.18	4 (0, 7)	2.11-7.26	1315.5	0.23
Anger–hostility	4 (2, 6)	4.15-5.34	5 (2, 8)	3.06-7.36	1480.5	0.66
Vigor	5 (2, 7)	4.06-5.02	3 (0, 5)	2.00-4.63	1220.0	0.11
Fatigue–inertia	6 (3, 9)	5.78-7.27	7 (3, 13)	5.58-10.42	1297.5	0.21
Confusion–bewilderment	5 (4, 7)	5.32-6.19	7 (4, 10)	5.50-8.92	1222.0	0.11

Mann–Whitney U test was used for statistics. Significant data are shown in bold

*CI* confidence interval, *GHQ* the 30-item version of the general health questionnaire, *POMS* the profile of mood states brief form

### Factors associated with students with suicidal ideation

Result of the logistic regression analysis using suicidal ideation of student as dependent variable is shown in Table 4. The GHQ suicidal depression subscale of students was not included in the regression analysis. The univariate logistic regression analyses revealed that five variables for students, including GHQ somatic symptoms, and POMS depression–dejection, anger–hostility, fatigue–inertia and confusion–bewilderment and four variables for caregivers, including their gender and suicidal depression from GHQ, and fatigue–inertia and confusion–bewilderment from POMS were significantly associated with suicidal ideation of students. Using a multivariate model with suicidal ideation of students as the dependent variable, students’ GHQ somatic symptoms, POMS confusion–bewilderment, and caregivers’ GHQ suicidal depression score were significantly associated with students’ suicidal ideation.

**Table 4:** Regression analysis of suicidal ideation of students

		Crude		Adjusted	
	*n* (%)	OR	95 % CI	OR	95 % CI
Sex					
Male	90 (48.6)	1.19	0.46-3.09		
GHQ					
General illness	6 (3.2)	1.79	0.20–16.17		
Somatic symptoms	11 (5.9)	6.06**	1.59–23.08	8.58*	1.39–53.03
Sleep disturbance	12 (6.5)	1.84	0.37–9.08		
Social dysfunction	2 (1.1)	9.17	0.55–152.90		
Anxiety and dysphoria	6 (3.2)	4.77	0.81–27.96		
POMS					
Tension–anxiety	5 (2.7)	6.39	0.99–40.96		
Depression–dejection	13 (7.0)	4.65*	1.28–16.92		
Anger–hostility	24 (13.0)	5.11**	1.77–14.74		
Vigor	99 (53.5)	1.22	0.47–3.18		
Fatigue–inertia	20 (10.8)	5.01**	1.65–15.23		
Confusion–bewilderment	29 (15.7)	6.57**	2.38–18.12	15.49**	3.79–63.26
Caregiver					
Mother	154 (83.2)	3.45*	1.24–9.65	7.29**	1.71–31.11
GHQ					
General illness	19 (10.3)	1.03	0.22–4.85		
Somatic symptoms	16 (8.6)	0.56	0.07–4.49		
Sleep disturbance	29 (15.7)	1.01	0.28–3.71		
Social dysfunction	6 (3.2)	4.77	0.81–27.96		
Anxiety and dysphoria	7 (3.8)	3.79	0.68–21.04		
Suicidal depression	11 (5.9)	14.86**	3.99–55.33	72.13**	12.60–412.96
POMS					
Tension–anxiety	11 (5.9)	2.05	0.41–10.29		
Depression–dejection	16 (8.6)	2.21	0.57–8.57		
Anger–hostility	24 (13.0)	1.30	0.35–4.82		
Vigor	100 (54.1)	1.97	0.71–5.42		
Fatigue–inertia	28 (15.1)	3.02*	1.04–8.78		
Confusion–bewilderment	31 (16.8)	3.45*	1.24–9.65		

Suicidal ideation of students as dependent variable. *OR* odds ratio, *CI* confidence interval. Adjusted for other factors in multivariate logistic regression analyses with stepwise elimination procedure at the *p* = 0.05 significance level for entry into the model

* *p* < 0.05, ** *p* < 0.01

## Discussion

This population-based cross-sectional survey revealed that suicide ideation was experienced in 10.3 % of junior high school students in the preceding weeks. We identified that not only mental state of students but also that of caregivers is associated with suicidal ideation of early teens. Comparing the mental state of students and their caregivers revealed more general illness and sleep disturbance in the caregivers, which are general trends for ageing and greater depressive and dejective mood than in the junior high school students. To the best of our knowledge, this is the first study to investigate the relationship between suicidal ideation and mental health of adolescents and those of caregivers using comparable batteries of questionnaire.

Previous studies demonstrated that the prevalence of suicidal ideation in the preceding 12 months in early teens was 9.8 % in Tanzania, 11.4 % in Malaysia, and 25.9 % in Kenya [24, 25]. The prevalence of lifetime suicidal ideation was reported to vary from 19 % to 44 % [26–30], which was equivalent to the prevalence of 40.4 % in Japan [31]. A randomized controlled trial by Gould et al. revealed that screening for mental health problems and suicide risk is an important component of youth suicide prevention efforts [20]. One of the causal factors for suicide is the risk from major depression, which has sharply increased between the ages of 12 and 16 years [32]. To prevent suicide, it is necessary to evaluate the mental states, including depressive symptoms, of junior and senior high school students.

### What the study adds to the existing evidence

From the logistic regression analysis, some scores were significantly associated with students’ suicidal ideation. Amongst them, the caregivers’ suicidal depression score had the highest odds ratio. Caregivers’ factors were also reported to be associated with the suicide risk of adolescents. Borowsky et al. examined risks and protective factors for suicide attempts in male and female adolescents and reported that closeness to parents is a protective factor against suicide in both sexes [33]. Parental psychological state may significantly change the number of suicide risks in adolescents [34]. Family discordance and negative relationships with parents have also been associated with an increased suicide risk in depressed adolescents [11]. Kwok et al. reported that higher levels of parent–adolescent communication were significantly correlated with lower levels of adolescent suicidal ideation [35]. Furthermore, mother–adolescent communication generally had a stronger effect than father–adolescent communication. These previous studies have demonstrated that familial factors are significantly associated with the risks for depression and suicide in adolescents.

In the present study, suicidal depression of caregivers was significantly associated with an increased risk of suicidal ideation in junior high school students after adjusting for other factors. We believe this is an important finding that suggests the need for professionals involved in suicide prevention paying attention to the mental state of caregivers, and the assessment using comparable batteries in an effective way of identifying mental health of both adolescents and their family in the community.

Although the caregivers’ suicidal depression score had the highest odds ratio, it was not possible to determine a causal link between the mental health of caregivers and suicidal ideation of their children from this cross-sectional study. The relationship between adolescents’ suicidal ideation and their mothers’ mental state is complicated, and this issue should be addressed in future investigations. Among the 17 subjects whose caregivers did not respond, poor mental state in the parents or a motherless and/or fatherless family may be present. In future research, it will be necessary to investigate familial background as well.

### Limitations

There were several other limitations in this study. This work was a population based study that surveyed all the junior high school students in the town. First, the sample size was still small not to deny the type II error, e.g. pseudo negative correlations of suicidal ideation; however, we consider it sufficient to indicate the differences between adolescents with and without suicidal ideation. We excluded one mentally retarded student from the study because the student had low IQ and could not answer the questionnaires properly, and in addition, we could not analyse 35 students whose caregivers did not take part in this study. The 35 students’ scores of GHQ and POMS showed no significant differences compared to the scores of 185 students. However, it could have influenced the study results. Since this survey was conducted only in a rural area, the results would not be generalized to the entire Japanese adolescent population. Second, the questionnaires used in this study evaluated mental state and behaviour only during recent weeks; therefore, it is not possible to assess mood changes and swings over time. Moreover, mood state measured by a few questionnaire questions is by itself part of the overall mental state. In addition, the POMS is validated over 15 years. Further longitudinal studies could support this work. Third, we had not controlled for confounding factors such as intelligence quotient, development disorders, mental disorders, and their domestic environment, including economic status. These factors could also influence risk factors for adolescent suicidal attempt and ideation. For understanding these factors, we plan to do interview to each student in the future.

## Conclusions

Suicidal ideation of children is associated with suicidal depression of their caregivers. Taking care of the caregiver’s mental state by medical professionals in addition to that of the adolescent may help early interventions aimed at reducing the risk of suicide in junior high school students. The assessment using comparable batteries for not only adolescents but also their caregivers in the community could be one of the strategies for the suicide prevention in adolescents.

## Abbreviations

GHQ, general health questionnaire; POMS, profile of mood states; TMD, total mood disturbance
